# Retrospective Benchmarking of Previously Modeled Coumarin–Pyrimidine Structures Against MDA-MB-231/MCF-10A Cellular Activity

**DOI:** 10.3390/biology15211999

**Published:** 2026-11-04

**Authors:** Veda B. Hacholli, Marcin Gackowski, Piotr Kośliński, Abhishek Kumar, Łukasz Szeleszczuk

**Affiliations:** 1 Department of Pharmaceutical Chemistry, Nitte College of Pharmaceutical Sciences (NCOPS), Nitte (Deemed to be University), Bengaluru 560064, Karnataka, India; vedab.hacholli@nitte.edu.in; 2 Department of Toxicology and Bromatology, Faculty of Pharmacy, Ludwik Rydygier Collegium Medicum in Bydgoszcz, Nicolaus Copernicus University in Torun, A. Jurasza 2 Street, 85089 Bydgoszcz, Poland; piotr.koslinski@cm.umk.pl; 3 Department of Pharmaceutical Chemistry, NGSM Institute of Pharmaceutical Sciences (NGSMIPS), Nitte (Deemed to be University), Mangaluru 575018, Karnataka, India; 4 Department of Organic and Physical Chemistry, Faculty of Pharmacy, Medical University of Warsaw, Banacha 1 Street, 02093 Warsaw, Poland; lukasz.szeleszczuk@wum.edu.pl

**Keywords:** coumarin–pyrimidine hybrids, molecular modeling, retrospective benchmarking, MDA-MB-231, MCF-10A, cytotoxicity, phenotypic endpoint, cross-endpoint transferability, physicochemical descriptors

## Abstract

This study retrospectively evaluates agreement between target-level molecular-modeling rankings from a previously published coumarin–pyrimidine library and an available cellular dataset for 14 experimentally evaluated compounds with documented structure assignments. Laboratory-reported MDA-MB-231 and MCF-10A IC_50_ values from multi-concentration MTT testing were compared with published AutoDock Vina scores using rank-based analyses. Compound **5e** had the lowest laboratory-reported MDA-MB-231 IC_50_ (12.4 ± 0.2 µM; *n* = 2) and the largest descriptive selectivity index (7.75); this ratio is specific to the MDA-MB-231/MCF-10A comparison and is not interpreted as evidence of general tumor selectivity. Docking rankings showed weak correspondence with cellular potency, and no docking–phenotype association remained statistically significant after Benjamini–Hochberg adjustment. No basic 2D descriptor showed a compelling adjusted association. Complete ^1^H/^13^C NMR and validated HRMS documentation, quantitative purity measurements, and the complete full-series MTT raw export are not available; structure-dependent and potency claims are therefore interpreted cautiously. Overall, the analysis demonstrates limited cross-endpoint transferability between target-level docking rankings and whole-cell activity in the MDA-MB-231/MCF-10A dataset, without establishing a validated structure–activity relationship, generalized TNBC activity, apoptosis induction, a BCL-2 mechanism, or translational efficacy.

## Simple Summary

Computer models can help prioritize molecules for experimental testing, but target-level predictions and whole-cell activity measure different aspects of biological behavior. Here, previously published docking rankings for coumarin–pyrimidine structures were compared retrospectively with laboratory-reported cytotoxicity data from MDA-MB-231 and MCF-10A cells. Compound **5e** had the lowest reported MDA-MB-231 IC_50_ and the largest descriptive MCF-10A/MDA-MB-231 IC_50_ ratio in the available dataset. Complete structural-characterization records, quantitative purity measurements, the complete full-series MTT raw export, and confirmatory biological replication were not available. Accordingly, the findings are interpreted descriptively rather than as definitive lead or mechanism validation. The central result is that target-level docking rankings did not directly reproduce the whole-cell potency ranking, supporting the need to benchmark computational prioritization against the biological endpoint used for compound selection.

## 1. Introduction

Breast cancer remains a major global cause of cancer morbidity and mortality and encompasses biologically distinct subtypes with different therapeutic vulnerabilities [1,2]. Triple-negative breast cancer (TNBC), defined by the absence of estrogen receptor, progesterone receptor, and HER2 overexpression/amplification, is clinically challenging because it lacks the endocrine and HER2-directed treatment options available for other breast cancer subtypes and frequently displays aggressive behavior, early relapse, and therapeutic resistance [2]. These features continue to motivate the development of small molecules capable of perturbing complementary pathways associated with survival, proliferation, DNA repair, and apoptosis.

Hybrid medicinal-chemistry strategies can combine pharmacophoric elements associated with different modes of target recognition. Coumarin derivatives occupy a broad pharmacological space and have repeatedly been explored as anticancer scaffolds, whereas pyrimidine motifs are common in kinase-directed and other oncology-focused small molecules [3,4]. Their combination, therefore, provides a chemically tractable platform for exploring polypharmacology and for testing how structural changes influence both target-level molecular recognition and phenotypic cellular responses [5].

In our previous work, a 64-member coumarin–pyrimidine library comprising hydroxy- and sulfhydryl-pyrimidine series was evaluated exclusively in silico against PARP-1, EGFR, HER2, and BCL-2 using molecular docking, 200 ns molecular dynamics simulations, MM-GBSA and MM-PBSA analyses, pharmacophore modeling, and in silico ADMET profiling [6]. That study identified target-specific computational leads and explicitly proposed subsequent synthesis and experimental evaluation. Importantly, the compounds experimentally investigated here belong to the same previously modeled chemical space; therefore, the earlier computational results are prior predictions rather than new results of the present work.

The availability of experimental cellular data now creates an opportunity distinct from simply repeating the original modeling workflow: computational rankings can be retrospectively examined against observed cellular activity. Such comparisons are informative because a docking score approximates the favorability of target–ligand interactions under a simplified model. In contrast, whole-cell cytotoxicity integrates permeability, intracellular exposure, target expression, metabolic stability, polypharmacology, and downstream cellular responses. Large comparative assessments have likewise shown that docking/scoring functions can be useful for pose generation or enrichment, yet are substantially less reliable as quantitative rankers of ligand affinity [7]. Therefore, imperfect agreement between target-specific scores and phenotypic potency is scientifically informative rather than intrinsically contradictory.

Accordingly, the present study retrospectively examines the available experimental follow-up data for 14 compounds (**5a**–**5n**) evaluated in MDA-MB-231 cells and non-tumorigenic MCF-10A cells using the structure assignments documented in the study records. The specific contribution is not the general observation that docking scores and cellular cytotoxicity represent different endpoints. Rather, it is a compound-level retrospective audit of an explicitly prospective, previously published multi-target modeling study against the subsequent experimental dataset generated for the corresponding chemical series. This structure-matched comparison identifies where individual computational priorities were retained or displaced after cellular testing and provides a transparent case study of model evaluation using predictions published before the experimental observations were available. Previously published molecular-modeling outputs are incorporated only as prior predictions, and simple 2D physicochemical descriptors provide a complementary target-independent context. Because the chemical-characterization documentation and biological replication are incomplete, the study focuses on descriptive cross-endpoint benchmarking and does not claim structural novelty, a validated structure–activity relationship, activity across TNBC as a disease class, or a confirmed molecular mechanism. Secondary Annexin V/PI and RT-qPCR records are retained only in the Supplementary Information for transparency and are not part of the primary evidentiary argument.

## 2. Materials and Methods

### 2.1. Study Design, Data Provenance, and Relationship to Previous Molecular Modeling

The present work retrospectively analyzes experimental follow-up data generated after the previously published in silico investigation of coumarin–pyrimidine hybrids [6]. The compound identities used in the analyses follow the assignments documented in the original study records for structures **5a**–**5n**. Complete original ^1^H/^13^C NMR records and validated HRMS metadata are not available for the full series; accordingly, the present manuscript does not claim independent re-characterization of every compound. Quantitative purity measurements are also unavailable. Structural characterization and quantitative purity are treated as distinct evidentiary questions: spectroscopic and accurate-mass data address structural assignment, whereas HPLC/UPLC, qNMR, or another validated quantitative method can document sample purity. The intended structures were already disclosed within the earlier computational library [6], and no claim of structural novelty is made in the present study. For retrospective benchmarking, each documented assignment was matched to the corresponding entry in the earlier library by molecular structure and substituent identity rather than by alphanumeric label. The detailed cross-reference is provided in Supplementary Information Table S1. Previously published docking, molecular-dynamics, free-energy, pharmacophore, and ADMET results are treated as prior predictions rather than as newly generated results.

Following a complete audit of the records and materials available to the authors for this revision, no additional validated analytical data, full-series biological raw datasets, or independently traceable material suitable for the requested reanalysis were identified. Generating the requested ^1^H/^13^C NMR, validated HRMS, quantitative purity, replicated cytotoxicity, or additional cell-based data would therefore require re-establishing the synthesis, analytical-characterization, and biological-testing workflows as a new prospective study. This practical limitation does not replace the requested evidence or resolve the reviewers’ underlying scientific concerns; it defines the boundary of the present retrospective revision, and the claims are restricted accordingly.

### 2.2. Synthetic Procedures and Sample Provenance

#### 2.2.1. Synthesis of 3-Acetyl-4-hydroxy-2*H*-chromen-2-one (**2**)

4-Hydroxycoumarin(Tokyo Chemical Industry (India) Pvt. Ltd., Chennai, India; CAS No. 1076-38-6) (1.0 mmol) was reacted with acetyl chloride (Tokyo Chemical Industry (India) Pvt. Ltd., Chennai, India; CAS No. 75-36-5) (1.0 mmol) in pyridine (Tokyo Chemical Industry (India) Pvt. Ltd., Chennai, India; CAS No. 110-86-1) (approximately 8 mL) in the presence of one drop of piperidine. The reaction was maintained at approximately 20 °C for 48 h. After completion, the product was isolated and recrystallized from ethanol. The represented structure corresponds to C_11_H_8_O_4_ (MW 204.18 g/mol). The reviewed source package did not contain a reconciled analytical record for intermediate 2; therefore, no experimental spectral assignment is claimed here. A review of 3-acetyl-4-hydroxycoumarin chemistry is cited as methodological context [8].

#### 2.2.2. Synthesis of 3-Acetyl-2-oxo-2*H*-chromen-4-yl methanesulfonate (**3**)

**Scheme 1:**
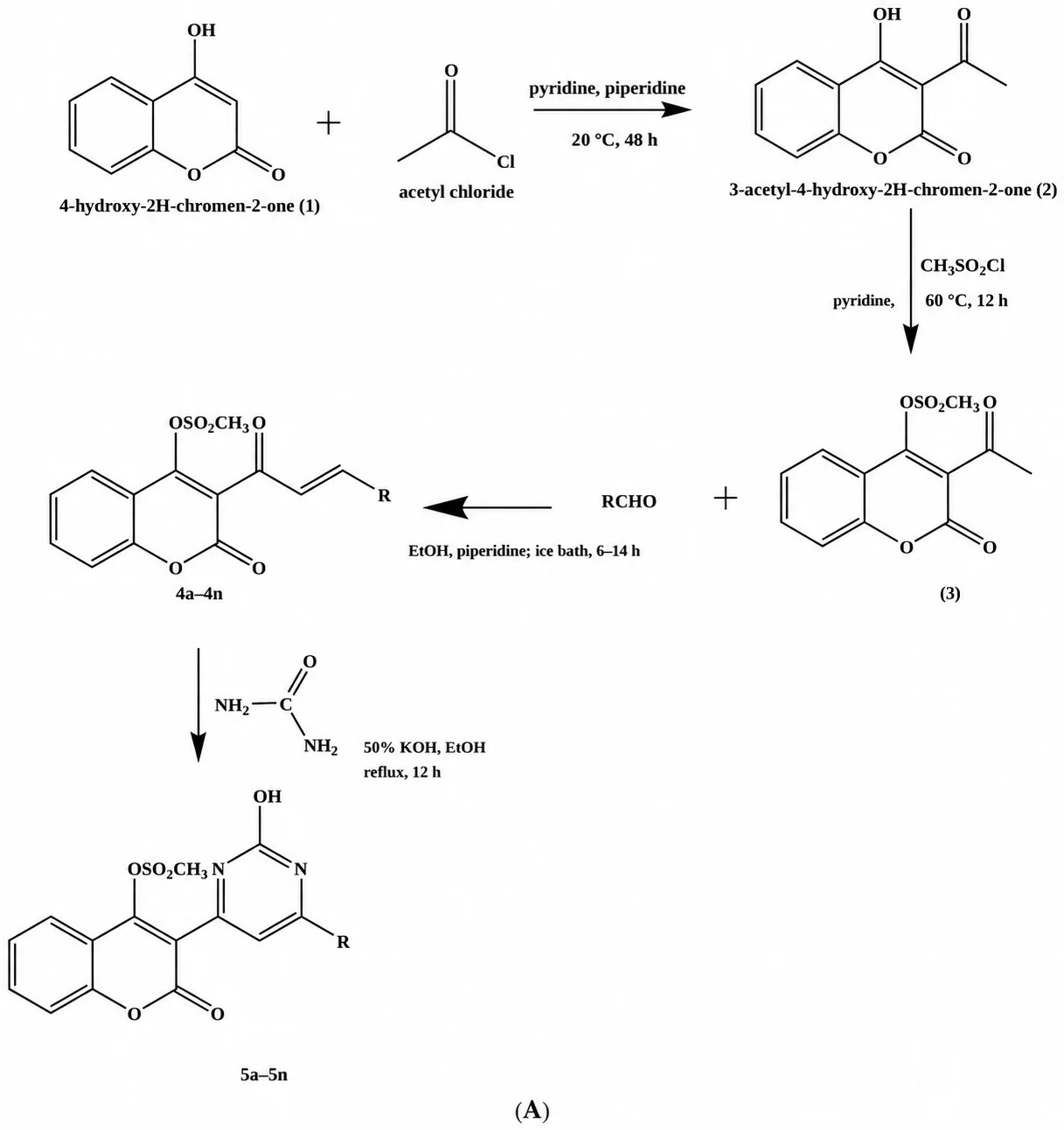

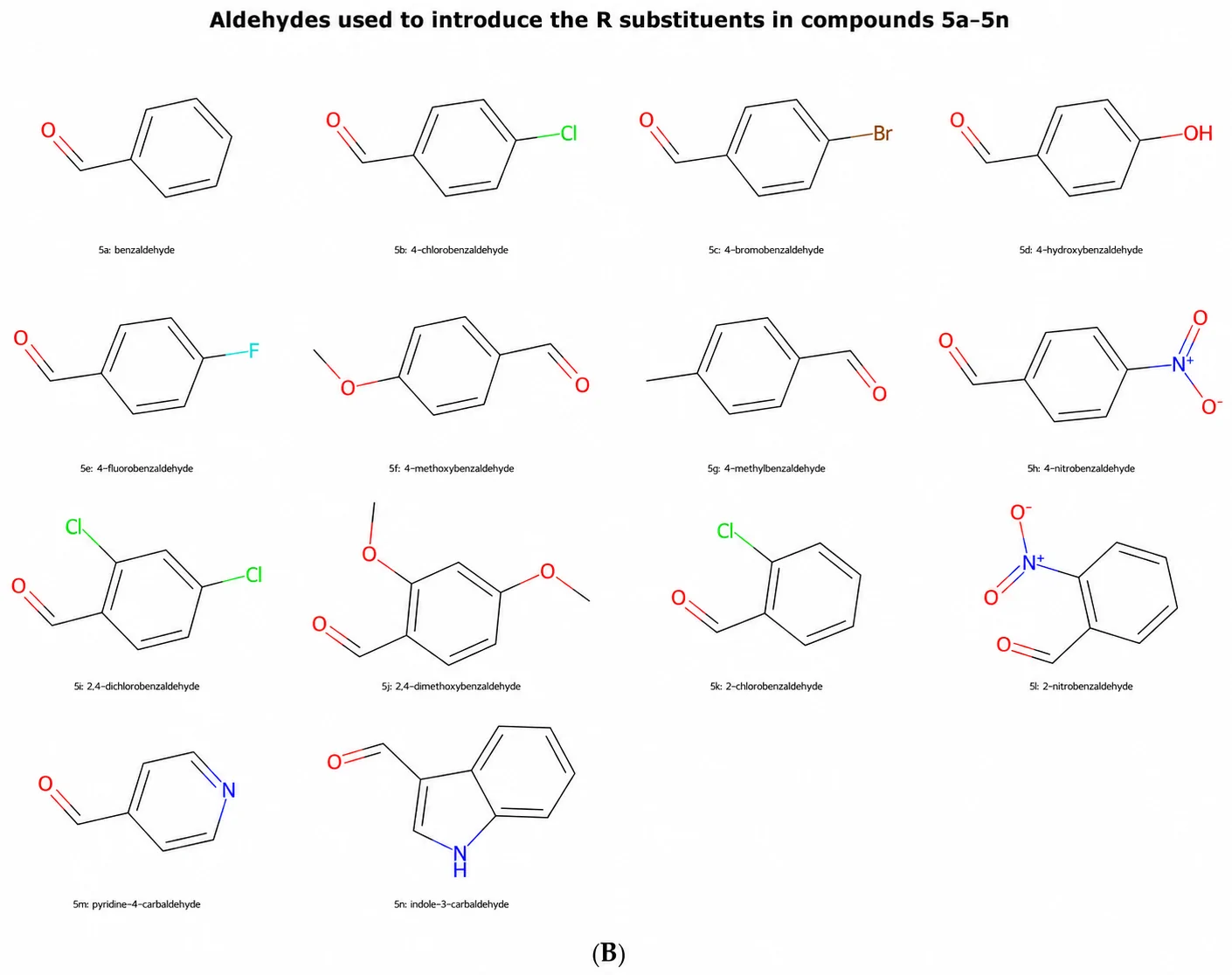
(**A**) Synthetic route to compounds **5a**–**5n**. Conditions: (i) acetyl chloride, pyridine, piperidine, approximately 20 °C, 48 h; (ii) methanesulfonyl chloride, pyridine, 60 °C, 12 h; (iii) the corresponding aldehyde, ethanol, piperidine, ice bath, approximately 6–14 h; (iv) urea, 50% KOH, ethanol, reflux, 12 h. Intermediates **4a**–**4n** were not isolated. (**B**) Structures of the aldehydes used to introduce the R substituents corresponding to compounds **5a**–**5n**.

Intermediate 2 (1.0 mmol) was treated with methanesulfonyl chloride (Tokyo Chemical Industry (India) Pvt. Ltd., Chennai, India; CAS No. 124-63-0) (1.0 mmol) in pyridine and heated at 60 °C for 12 h under a reflux condenser (Borosil Scientific Limited, Mumbai, India). The product was purified by recrystallization from ethanol to give the material designated intermediate 3 in 75% isolated yield. The intended structure shown in Scheme 1 corresponds to C_12_H_10_O_6_S (MW 282.27 g/mol; neutral exact mass 282.0198). The available mass-spectrum record is insufficient for definitive independent structural confirmation and is therefore not used as such. A precedent for coumarin sulfonylation is cited as methodological context [9].

#### 2.2.3. Sequential Formation of Chalcone Intermediates (**4a**–**4n**)

Intermediate 3 (corresponding to 1.0 mmol) was reacted with the appropriate aromatic or heteroaromatic aldehyde (1.0 mmol) in ethanol in the presence of piperidine (Tokyo Chemical Industry (India) Pvt. Ltd., Chennai, India; CAS No. 110-89-4) while the reaction vessel was maintained in an ice bath; the exact internal temperature was not recorded. Reaction progress was monitored by thin-layer chromatography, and the available experimental record reports reaction times of approximately 6–14 h, depending on the aldehyde. The chalcone intermediates **4a**–**4n** were not isolated or purified separately; the reaction mixture was carried directly into the subsequent cyclization step. Accordingly, isolated yields are not assigned to **4a**–**4n** in the present manuscript. Related coumarin–chalcone synthesis has been reported previously [10].

#### 2.2.4. Cyclization to Hydroxy-Pyrimidine–Coumarin Derivatives **5a**–**5n**

Without isolation of intermediates **4a**–**4n**, the crude material generated from the preceding aldehyde-condensation step (derived from 1.0 mmol of intermediate 3 on a theoretical basis) was taken forward to cyclization with urea (Tokyo Chemical Industry (India) Pvt. Ltd., Chennai, India; CAS No. 57-13-6) (3.0 mmol) in 50% KOH (Tokyo Chemical Industry (India) Pvt. Ltd., Chennai, India; CAS No. 110-89-4) (4 mL) and ethanol (10 mL) under reflux for 12 h. After completion, the reaction mixture was cooled and poured into ice-cold water. The precipitated products were collected by filtration, washed with cold water, and recrystallized from ethanol to afford derivatives **5a**–**5n**. Related chalcone-to-pyrimidine cyclization has been reported previously [11].

### 2.3. Sample Labels, Assigned Structures, Theoretical Molecular Data, and Available Analytical Records

Compound labels **5a**–**5n** and the corresponding documented substituent assignments are listed in Table 1. The available records report isolated yields of 62–79% for the final derivatives and melting-point ranges spanning 225–272 °C. Molecular formulas, average molecular weights, neutral monoisotopic masses, and theoretical [M + H]^+^ values were calculated from the assigned structures and are reported separately from experimental mass-spectrometric observations. The analytical facility was reported to use a Waters ACQUITY platform (Waters Corporation, Milford, MA, USA) with MassLynx software (version 4.1; Waters Corporation, Milford, MA, USA) in positive-ion electrospray mode; however, the complete mass-spectrometer/analyzer model, calibration/error information, and raw accurate-mass metadata were not available. The supplied PDFs are therefore described as positive-ion ESI-MS records rather than as validated HRMS data. No complete original ^1^H/^13^C NMR series or elemental analysis was available, and no quantitative purity measurements by HPLC/UPLC, qNMR, or an equivalent method were retained. Structural characterization and quantitative purity are separate evidentiary questions, and both are incompletely documented in the available records. The manuscript therefore avoids purity-qualified or identity-dependent SAR claims. A documented file-label reversal between **5d** and **5e** was corrected following author clarification, and only the VB2 urea-series page of the merged **5b** ^13^C PDF is relevant to the present **5b** sample; further details are summarized in the Supplementary Information.

### 2.4. Cell Culture

MDA-MB-231 human TNBC cells were obtained from NCCS (Pune, India) and maintained in DMEM (Gibco, Thermo Fisher Scientific, Waltham, MA, USA) supplemented with 10% fetal bovine serum (FBS; Gibco, Thermo Fisher Scientific, Waltham, MA, USA) and 1% antibiotic–antimycotic solution (Gibco, Thermo Fisher Scientific, Waltham, MA, USA). Cells were cultured at 37 °C in a humidified atmosphere containing 5% CO_2_ and were subcultured approximately every 2 days; passage P49 was reported for the experiments. According to the available experimental record, MCF-10A non-tumorigenic mammary epithelial cells were obtained from ATCC and maintained in DMEM/F12 (Gibco, Thermo Fisher Scientific, Waltham, MA, USA) supplemented with 5% fetal bovine serum, approximately 20 ng/mL EGF, 10 µg/mL insulin, 0.5 µg/mL hydrocortisone, and 1% antibiotic–antimycotic solution under the same incubator conditions; passage P16 was reported. Independent records documenting cell line authentication or mycoplasma testing were not available in the study package under review.

### 2.5. MTT Cytotoxicity Assay

Cells were seeded in 96-well plates at 15,000 cells per well and allowed to adhere before treatment. A general assay record lists a nominal treatment range of 3.125–100 µg/mL for 24 h, with compounds dissolved in DMSO (Tokyo Chemical Industry (India) Pvt. Ltd., Chennai, India; CAS No. 67-68-5). Subsequent author clarification identified the negative control as the DMSO vehicle; however, the exact final DMSO concentration was not documented in the available records and is not reconstructed here. Doxorubicin (Tokyo Chemical Industry (India) Pvt. Ltd., Chennai, India; CAS No. 25316-40-9) was used as the positive-control drug. Following exposure, MTT (Tokyo Chemical Industry (India) Pvt. Ltd., Chennai, India; CAS No. 298-93-1) reagent was added, and metabolically active cells were allowed to reduce MTT to formazan; the formazan product was solubilized, and absorbance was measured at 570 nm. Cell viability was normalized to the corresponding control. The mass-to-molar conversion used for compound-specific reporting follows µM = [concentration (µg/mL) × 1000]/molecular weight (g/mol), using the theoretical molecular weights in Table 1. The complete compound-by-compound concentration series is not retained, so this conversion and the curve fitting cannot be independently audited for every individual concentration point. The outsourced laboratory confirmed that compounds **5a**–**5n** were each evaluated using a multi-concentration procedure to determine IC_50_ values. No compound-specific concentration-wise reports or complete full-series plate-reader export are available in the submitted records. Consequently, dose–response curves, goodness-of-fit measures, and curve-fit confidence intervals cannot be independently reconstructed for every compound from the available material. The exact MTT reagent concentration/volume, incubation time with MTT, solubilization reagent, and final DMSO concentration are not available in the current records [12]. Because MTT quantifies tetrazolium-reducing metabolic activity, and no orthogonal direct cell-count, membrane-integrity, or ATP-based assay was performed, the endpoint is not interpreted as a direct measurement of cell number.

For compound **5e**, the outsourced laboratory reported an IC_50_ of 12.4 ± 0.2 µM, with ±0.2 indicating the standard deviation of two independent measurements (*n* = 2). The laboratory further confirmed that all reported **5a**–**5n** IC_50_ values were obtained from multi-concentration measurements. Because the complete full-series plate-reader export is unavailable, the concentration–response calculations cannot be independently reconstructed for every compound. The IC_50_ values are therefore treated as laboratory-reported descriptive estimates, and no inferential comparison between compounds is performed. No claim is made that the observed potency ranking would remain unchanged after additional independent experiments. Single-point viability values are not reported because the corresponding test concentration was not documented.

### 2.6. Selectivity Index

For compounds with reported IC_50_ values in both cell models, a descriptive selectivity index (SI) was calculated as IC_50_(MCF-10A)/IC_50_(MDA-MB-231). SI values are reported as descriptive ratios and do not substitute for formal propagation of uncertainty from the underlying IC_50_ estimates. Because each ratio is derived from one malignant and one non-tumorigenic cell line, it is considered specific to this cell-model comparison and is not interpreted as evidence of generalized tumor selectivity.

### 2.7. Retrospective Computational–Experimental Benchmarking

Previously published AutoDock Vina (version 1.1.2) scores for structurally corresponding compounds were extracted from Table 2 of our earlier computational study [6] and were treated as prior predictions rather than as newly generated docking data. The primary retrospective benchmark included 13 compounds (**5a**–**5m**) for which unambiguous structure-level correspondence to the previously modeled library could be assigned. Compound **5n** was excluded from the primary analysis because the printed SMILES for the intended indole analog in the earlier publication is inconsistent with the structure assigned to **5n** in the present study; a sensitivity analysis additionally considered the intended structure-level correspondence for **5n**. For each target (PARP-1, EGFR, HER2, and BCL-2), Spearman rank correlations were calculated between the previously published docking score and the reported MDA-MB-231 IC_50_ value. The same target-specific scores were also compared with the descriptive selectivity index (SI). Because more negative docking scores indicate stronger predicted binding, whereas larger SI values indicate greater differential cytotoxicity, a negative docking–SI correlation represents directional concordance. To summarize multi-target ranking without averaging absolute Vina scores across different protein pockets, compounds were ranked separately within each target (rank 1 = most favorable docking score), and the mean of the four within-target ranks was compared with the experimental MDA-MB-231 potency rank. Kendall τb was used to conduct a rank-based sensitivity analysis of the principal associations. Nine exploratory rank-correlation tests were considered in the primary benchmark (four targets versus IC_50_, four targets versus SI, and the cross-target mean-rank comparison); Benjamini–Hochberg false-discovery-rate adjustment was therefore applied across these tests. Because the BCL-2 docking-versus-SI comparison produced the strongest nominal association, a post hoc leave-one-out sensitivity analysis was also performed by omitting each compound in turn and recalculating Spearman ρ. Raw *p*-values and adjusted q-values are interpreted descriptively because this is a retrospective, hypothesis-generating analysis, and the complete full-series raw MTT export is unavailable. The complete row-level benchmark, structure cross-reference, within-target ranks, correlation summary, and leave-one-out results are provided in Supplementary Information Tables S1, S3, S4, and S4A.

### 2.8. Supplementary Exploratory Biological Records

The available Annexin V/PI and RT-qPCR records have been removed from the primary Methods and Results and are provided only in the Supplementary Information for transparency. The Supplementary Information contains the available procedures, source-data limitations, descriptive values, and the relevant methodological references [13,14,15,16]. Because the Annexin V/PI record derives from one independent experiment and the RT-qPCR record from two biological replicates with incomplete control and quality documentation, neither dataset is used to support the primary conclusions, apoptosis induction, pathway activation, or target engagement.

### 2.9. Two-Dimensional Physicochemical Descriptor Analysis

To provide a target-independent molecular context, a reproducible 2D descriptor analysis was performed without relying on unavailable target-prediction raw files.

Canonical structures for **5a**–**5n** were standardized from the molecular structures assigned to the experimental sample labels (Supplementary Information Table S2) and analyzed using RDKit 2025.09.4 [17]. The following basic 2D descriptors were calculated: molecular weight (MW), Crippen cLogP, topological polar surface area (TPSA), hydrogen-bond acceptor count (HBA), hydrogen-bond donor count (HBD), rotatable-bond count, fraction of sp3 carbon atoms (fractionCSP3), and aromatic-ring count. For each descriptor, Spearman’s rank correlation was calculated against the laboratory-reported MDA-MB-231 IC_50_ and the descriptive selectivity index. Kendall τb was calculated as a sensitivity measure. Because eight descriptors were tested against two outcomes, Benjamini–Hochberg false-discovery-rate adjustment was applied across 16 exploratory descriptor–phenotype comparisons. This analysis was intended to determine whether simple molecular size, lipophilicity, polarity, hydrogen-bonding capacity, flexibility, or aromaticity could account for the experimental ranking; it was not treated as a predictive QSAR model.

### 2.10. Statistical and Descriptive Analysis

Where replication and source data permit, continuous results are summarized using mean and dispersion measures appropriate to the underlying records. Laboratory-reported MTT IC_50_ values are summarized as mean ± SD from two independent experiments (*n* = 2), and selectivity indices are calculated descriptively from those values. No inferential comparison between compounds is performed. The secondary Annexin V/PI and RT-qPCR records are retained only in the Supplementary Information and are not included in the primary inferential analysis. Retrospective docking–phenotype and descriptor–phenotype associations are assessed using rank-based correlation coefficients with multiplicity adjustment as described above. Secondary statistical analyses were executed in Python 3.13.5 using SciPy 1.17.0 and statsmodels 0.14.6; figures were generated with Matplotlib 3.10.8. The row-level data and summary statistics required to reconstruct the reported computational analyses are provided in the Supplementary Information.

### 2.11. Supplementary Data and Reproducibility

The Supplementary Information provides the structure-level cross-reference and canonical inputs (Tables S1 and S2), the row-level retrospective docking dataset and correlation summaries (Tables S3, S4 and S4A), the single-experiment Annexin V/PI source record (Table S4B), RT-qPCR methods, Cq values, and replicate-level calculations (Tables S5 and S5A), RNA quantification data (Table S6), primer information (Table S7), the 2D descriptor matrix and descriptor–phenotype correlations (Tables S8 and S8A), amplification profiles (Figure S1), gene-specific melt-curve outputs (Figure S2), and a concise summary of the available analytical records. Previously published docking values are retained unchanged as prior predictions. Observed m/z values are transcribed from the supplied positive-ion ESI-MS PDFs without numerical adjustment. The documented **5d**/**5e** file-label reversal and the **5b**/VB2 ^13^C clarification are incorporated as author-provided source-record corrections; unresolved analytical records are not used as definitive structure-confirmation evidence.

### 2.12. Use of Generative AI-Assisted Tools

ChatGPT (OpenAI; GPT-5.6 Sol) was used during manuscript preparation solely for language editing (grammar, spelling, punctuation, and stylistic refinement) and to assist with selected graphical elements. The authors produced the scientific content, data analyses, and interpretation. All outputs used for these limited purposes were reviewed and edited by the authors, who take full responsibility for the final manuscript.

## 3. Results

### 3.1. Evaluated Compound Series and Available Analytical Records

The experimentally evaluated series comprised 14 compounds with documented assignments to hydroxy-pyrimidine–coumarin structures differing in the aryl or heteroaryl substituent (Table 1). The final products were reported as yellow-to-orange crystalline solids with isolated yields of 62–79% and melting-point ranges of 225–272 °C. For retrospective benchmarking, 13 documented assignments could be unambiguously matched to entries in the previously modeled library. Molecular formulas, average molecular weights, neutral exact masses, and theoretical [M + H]^+^ values were calculated from the assigned structures. The available positive-ion ESI-MS PDFs and partial ^13^C record are retained as supporting analytical records but do not constitute a complete structural-characterization package or a quantitative purity assessment. Accordingly, structure-dependent interpretation is kept cautious, and no validated SAR is claimed.

### 3.2. Laboratory-Reported Cytotoxicity and Descriptive Selectivity

The outsourced laboratory confirmed that all compounds **5a**–**5n** were evaluated by multi-concentration MTT measurements. Within the available laboratory-reported dataset, compound **5e** displayed the lowest MDA-MB-231 IC_50_ (12.4 ± 0.2 µM), followed by **5m** (13.8 ± 0.3 µM) and **5h** (14.8 ± 0.2 µM). Most other compounds showed IC_50_ values in the approximately 20–25 µM range. In MCF-10A cells, the reported IC_50_ for **5e** was 96.1 ± 2.2 µM, corresponding to a descriptive SI of approximately 7.75. The next highest descriptive SI values were observed for **5m** (approximately 6.77) and **5h** (approximately 5.72). These values identify **5e** as the most active compound in the available MDA-MB-231 dataset; however, the ranking remains descriptive because each IC_50_ is based on two independent experiments, and complete chemical characterization, quantitative purity measurements, and the full-series raw MTT dataset are unavailable. The SI is specific to the MDA-MB-231/MCF-10A comparison and is not interpreted as general tumor selectivity. The laboratory-reported values are summarized in Table 2.

### 3.3. Cross-Endpoint Benchmarking of Prior Molecular-Modeling Rankings

**Figure 1:**
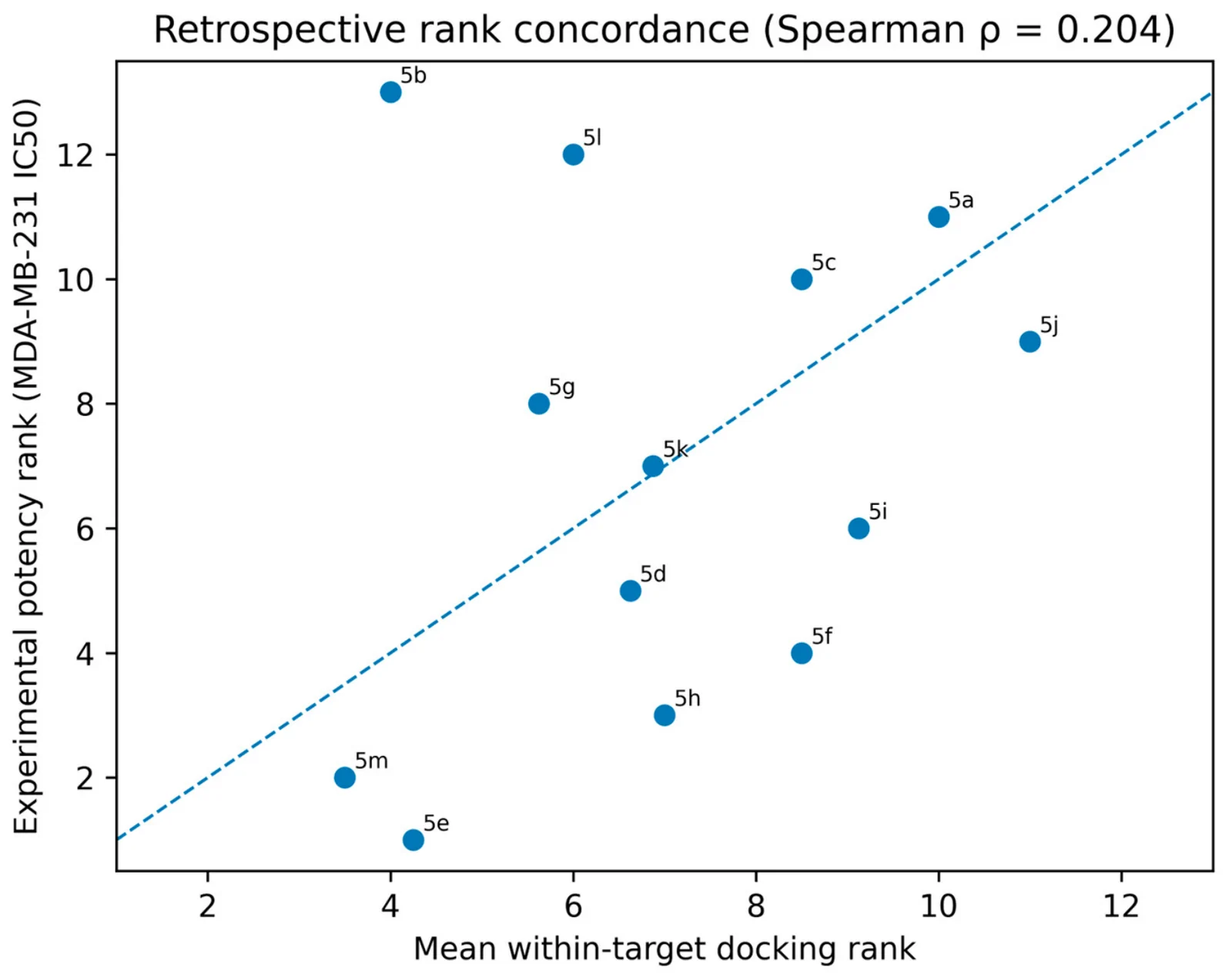
Retrospective comparison of mean within-target docking rank with laboratory-reported MDA-MB-231 potency rank for 13 compounds with unambiguous structural correspondence to the prior library [6]. Ranks were calculated within each target (rank 1 = most favorable Vina score) and averaged; the experimental rank used the lowest IC_50_ as rank 1. The dashed line indicates perfect concordance (Spearman ρ = 0.204; nominal *p* = 0.505).

The primary retrospective benchmark included 13 experimentally tested compounds with unambiguous correspondence to the previously modeled library. Target-specific docking scores showed little rank concordance with the reported MDA-MB-231 potency for PARP-1 (Spearman ρ = 0.017, nominal *p* = 0.956), EGFR (ρ = 0.042, *p* = 0.893), or HER2 (ρ = 0.081, *p* = 0.793). BCL-2 docking showed a moderate but non-confirmatory association with MDA-MB-231 IC_50_ (ρ = 0.492, *p* = 0.087). When the four target-specific ranks were combined by averaging within-target rank positions, the resulting multi-target rank likewise showed weak correspondence with the experimental potency rank (ρ = 0.204, *p* = 0.505; Kendall τb = 0.142, *p* = 0.501; Figure 1). This comparison is not interpreted as a test of whether docking “works” or “fails”; rather, it quantifies cross-endpoint transferability between target-level computational rankings and a whole-cell phenotypic readout that also depends on permeability, intracellular exposure, metabolism, target abundance, off-target activity, and downstream responses. The complete primary dataset and rank calculations are provided in Supplementary Information Table S3.

At the compound level, the phenotypic ranking did not mirror the computational ordering. Compound **5e**, which ranked first by MDA-MB-231 potency, had a mean within-target docking rank of 4.25, whereas **5m** ranked second experimentally and had the most favorable mean docking rank among the primary benchmark compounds (3.50). Compound **5h** ranked third experimentally despite a less favorable mean docking rank (7.00). These rank displacements emphasize that retrospective benchmarking evaluates translation between different endpoints rather than asking whether a docking score reproduces a whole-cell assay.

Across the nine primary exploratory docking–phenotype comparisons, no association remained statistically significant after Benjamini–Hochberg adjustment. The smallest adjusted q value occurred for the previously published BCL-2 docking score versus the descriptive selectivity index (Spearman ρ = −0.680, nominal *p* = 0.011; Kendall τb = −0.522, *p* = 0.018; Benjamini–Hochberg q = 0.095; Supplementary Information Table S4). A sensitivity analysis that additionally included 5n using its intended structure-level correspondence retained the same direction and a similar magnitude (Spearman ρ = −0.595, nominal *p* = 0.025). In a post hoc leave-one-out analysis of the 13-compound primary set, the inverse association also retained its direction after omission of every individual compound (Spearman ρ range, −0.769 to −0.605; Supplementary Information Table S4A). These robustness checks do not change the multiplicity-adjusted primary result. Because direct BCL-2 binding or target engagement was not measured, the observation is retained only as a future hypothesis and is not assigned mechanistic priority.

### 3.4. Two-Dimensional Physicochemical Descriptor Context

Basic 2D descriptors were calculated for all 14 assigned structures to test whether simple molecular properties showed monotonic relationships with the reported cellular ranking. Across MW, cLogP, TPSA, HBA, HBD, rotatable-bond count, fractionCSP3, and aromatic-ring count, no descriptor showed a compelling association with MDA-MB-231 IC_50_ or SI after Benjamini–Hochberg adjustment across the 16 descriptor–phenotype tests. The largest nominal trend was an inverse relationship between MW and SI (Spearman ρ = −0.506, nominal *p* = 0.065), which remained non-significant. These exploratory descriptor analyses are not used to assign a structural cause to the ranking of **5e** or to infer a structure–activity relationship. The full descriptor matrix and rank-correlation results are provided in Supplementary Information Tables S8 and S8A.

## 4. Discussion

This study is best interpreted as a retrospective comparison of a previously published computational discovery program with subsequent laboratory-reported cellular data. The earlier work established a molecular-modeling landscape for 64 coumarin–pyrimidine structures across four breast-cancer-relevant targets [6]. In the available MDA-MB-231/MCF-10A dataset, compound **5e** has the lowest reported MDA-MB-231 IC_50_. Given the incomplete chemical characterization and quantitative purity documentation and the limited biological replication, **5e** is described as the most active compound in the available dataset rather than as a validated medicinal-chemistry lead.

The retrospective benchmark makes the distinction between target-level modeling and whole-cell phenotype explicit. Across the 13 unambiguously mapped compounds, prior docking ranks for PARP-1, EGFR, and HER2 showed essentially no correspondence with the reported MDA-MB-231 potency, and BCL-2 showed only a moderate, non-confirmatory relationship with IC_50_. A cross-target average of within-target ranks was similarly weakly associated with experimental potency (ρ = 0.204). The result is therefore inconsistent with a simple claim that docking rank directly predicted cytotoxic potency. This is not unexpected: docking scores approximate molecular recognition in a defined protein pocket, whereas MTT-based cytotoxicity integrates permeability, solubility, intracellular exposure, metabolism, target abundance, off-target pharmacology, and downstream network responses. The ranking displacements are consequently informative rather than merely negative, because they define where molecular-level prioritization did and did not translate to phenotype. This interpretation is consistent with prior large-scale evaluations showing limited reliability of docking scores for quantitative affinity ranking [7].

The specific contribution of the retrospective benchmark lies in its temporal and structure-level design. The target-level predictions were published before the cellular observations were available, and the present analysis maps those prior predictions to the subsequently tested, corresponding chemical series at the individual-compound level. This permits an audit of where prospective priorities were retained or displaced after whole-cell testing, rather than a post hoc demonstration that docking and cytotoxicity are generally different endpoints. The earlier computational study selected target-specific complexes for detailed dynamic analysis based on docking and interaction profiles [6], whereas the available cellular dataset ranks **5e** first by MDA-MB-231 IC_50_ and **5m** second. The difference is not interpreted as a failure of molecular modeling; it is a compound-resolved case study showing why prospective computational prioritization must ultimately be benchmarked against the biological readout used for selection.

These findings also sit within a rapidly evolving TNBC treatment landscape. Contemporary reviews emphasize that immune checkpoint inhibition, PARP inhibition, antibody–drug conjugates, and other targeted strategies have expanded therapeutic options, yet benefit remains biologically stratified and resistance, heterogeneity, and the need for additional tractable small-molecule approaches remain important challenges [18,19,20]. A recent systematic review of anti-TNBC medicinal chemistry likewise highlighted nitrogen-containing heterocycles, halogenated analogs, and structure-guided optimization across targets including EGFR and PARP1, while emphasizing the need to link in silico prioritization to experimental validation [21]. In parallel, recent coumarin–chalcone work in breast cancer models has continued to combine synthesis, cytotoxicity testing, and molecular docking, illustrating the broader movement toward experimentally anchored interpretation of computationally prioritized scaffolds [22].

Breast-cancer heterogeneity also limits interpretation of a ratio derived from one malignant cell line and one non-tumorigenic comparator. Network-based biomarker analysis has highlighted prognostic and molecular heterogeneity across breast-cancer datasets [23], while a multicenter study of HER2 testing demonstrated substantial between-cohort variation even within a clinically defined breast-cancer subgroup [24]. A recent TNBC nanoplatform study combined MDA-MB-231 experiments with immune co-culture and in vivo evaluation [25], illustrating a substantially broader validation framework than is available here. Computationally generated hypotheses in a different molecularly stratified cancer context have likewise been explicitly framed as requiring subsequent in vitro, in vivo, and pharmacological validation [26]. These studies do not validate the present compounds, but they reinforce why the reported SI of 7.75 should be regarded only as a cell-model-specific descriptive ratio and why broader TNBC relevance requires additional models.

The coumarin–pyrimidine series offers a chemically tractable scaffold for future investigation, but the present dataset does not demonstrate therapeutic superiority over established or emerging TNBC approaches. Studies of a metal–organic-framework/MnO2 system co-delivering doxorubicin and a DNAzyme illustrate how delivery engineering and combination treatment can be evaluated within a controlled platform [27], whereas a study of chrysotoxine in cervical cancer combined phenotypic assays with ferroptosis and PI3K/AKT/mTOR analyses [28]. A review of immunotherapy in the ovarian-cancer microenvironment further illustrates that treatment interpretation depends on disease-specific immune and stromal context [29]. These publications concern different therapeutic modalities and, in two cases, different cancer types; they are cited as contextual examples of the evidence needed for delivery, mechanism, and microenvironment claims, not as direct support for compound **5e**.

The present retrospective design is also consistent with current thinking on target-based versus phenotypic drug discovery. Recent analyses argue that disease-relevant phenotypic screening and target-based approaches are complementary rather than mutually exclusive, because pathway knowledge and target engagement do not, by themselves, guarantee translation into a cellular therapeutic phenotype [30,31]. Similarly, structure-based virtual screening remains valuable for chemical-space triage. Still, recent methodological discussions emphasize hit-rate context, pose-selection uncertainty, training-set bias, and limited generalizability of scoring functions as reasons to benchmark computational predictions against independent experimental endpoints whenever possible [32,33].

Among the exploratory docking–phenotype comparisons, the smallest adjusted q value occurred for BCL-2 docking versus the descriptive selectivity index. Although the nominal Spearman association was ρ = −0.680 (*p* = 0.011), the Benjamini–Hochberg-adjusted q value was 0.095 and therefore did not meet the prespecified q < 0.05 threshold. The direction persisted when 5n was included in a sensitivity mapping and in each leave-one-out analysis, but these post hoc checks do not convert the multiplicity-adjusted result into confirmatory evidence. BCL-2 is therefore not assigned mechanistic priority,. Direct target-engagement experiments would be required to evaluate that hypothesis.

Cytotoxicity, a descriptive SI, and target-specific activity are separate evidentiary levels. Mechanistic studies in other cancer systems have linked molecular regulation to phenotype by combining perturbation or rescue experiments with protein-level, interaction, degradation, and functional assays—for example, studies of MAEL-dependent metabolic reprogramming in breast cancer [34] and PSMD12-dependent CDK1 stabilization in hepatocellular carcinoma [35]. Although those pathways differ from the targets considered here, they illustrate why docking alone cannot establish target engagement or causal pathway dependence. Direct binding or target-engagement measurements, protein-level pathway readouts, and perturbation/rescue experiments would be required to connect any proposed target with the observed cellular phenotype.

Among the currently reported cellular data, compound **5e** has the lowest MDA-MB-231 IC_50_ and the largest descriptive IC_50_ ratio relative to MCF-10A. The complementary 2D descriptor analysis did not identify a simple monotonic relationship between the reported phenotype and MW, cLogP, TPSA, hydrogen-bond counts, flexibility, fractionCSP3, or aromatic-ring count after multiplicity adjustment. Given the limitations of the available chemical-characterization records, these descriptor results are used only as contextual analyses and are not interpreted as evidence that a particular substituent or molecular property causes the observed ranking.

For descriptive completeness, the available dataset ranks compounds **5e**, **5m**, and **5h** as the three compounds with the lowest reported MDA-MB-231 IC_50_ values. This ordering is not used to infer a quantitative or qualitative structure–activity relationship. Without complete structural characterization and quantitative purity data for the tested materials, causal identity-dependent SAR statements would exceed the support of the available records. The observed ranking is instead treated as a basis for prioritizing future re-synthesis, full characterization, and independent retesting.

The MTT assay adds a further endpoint-specific limitation. Tetrazolium reduction depends on cellular metabolic activity and can change without a proportional change in cell number. Because no orthogonal direct-counting, membrane-integrity, clonogenic, or ATP-based assay was available, the reported IC_50_ values should be interpreted as MTT-derived metabolic cytotoxicity estimates rather than independently confirmed loss of viable cell number. An MTT-derived cytotoxicity estimate also cannot distinguish between specific cell-death pathways, including caspase-3-associated apoptosis and pyroptosis [36]. Similarly, research on AS1411-functionalized albumin nanoparticles for nucleolin-targeted realgar delivery in TNBC illustrates the importance of complementary assessments of cellular uptake, biological mechanisms, and antitumor effects [37]. These studies provide methodological context but do not establish the mechanisms underlying the activity of compound **5e**.

The same caution applies to target-prediction and network-level interpretation. Network-based drug-discovery methods and network pharmacology can organize multi-target hypotheses and prioritize biologically connected mechanisms. Still, their outputs depend strongly on source-database coverage, target-prediction quality, and transparent reporting of the compound–target edges used to build the network [38,39]. Large-scale evaluation of reverse target prediction has likewise shown that such methods can provide useful enrichment for phenotypic screening and polypharmacology, while underscoring the need for application-oriented benchmarking and high-quality reference data [40]. Accordingly, any future network analysis of **5e** should be treated as hypothesis-generating and should not be presented as direct target engagement. This distinction is particularly important for BCL-2-related hypotheses because apoptosis regulation depends on interactions among BCL-2 family members, including those mediated by their C-terminal sequences [41].

Several limitations directly constrain interpretation of the present dataset. First, complete original ^1^H/^13^C NMR records and validated HRMS metadata are unavailable for the full series, and no quantitative purity measurements by HPLC/UPLC, qNMR, or an equivalent method are available in the study records. These are distinct limitations: the former restricts independent structural verification, whereas the latter prevents a quantitative purity assessment. The available ESI-MS and partial ^13^C records provide supporting documentation but do not eliminate either limitation; the documented **5d**/**5e** file-label reversal further warrants caution. Second, although the outsourced laboratory confirmed multi-concentration MTT testing for every compound, the complete full-series plate-reader export is unavailable, the final DMSO concentration is not documented, each reported IC_50_ is based on only two independent experiments, and the ranking has not been validated with an orthogonal viability method. Third, the secondary Annexin V/PI and RT-qPCR records are retained only in the Supplementary Information: the former derives from one independent experiment with substantial control apoptosis, whereas the latter contains two biological replicates and incomplete efficiency, inhibition, cycling, RNA-quality, and vehicle-matching documentation. These secondary records are not used to support the primary conclusions or any mechanistic inference. Fourth, only one TNBC cancer cell line was evaluated; MDA-MB-231 cannot represent the biological heterogeneity of TNBC, and no inference is made about activity across TNBC as a disease class. Finally, no organoid, in vivo efficacy, pharmacokinetic, biodistribution, safety, or tumor-microenvironment experiment was performed. Following a complete audit of the records and materials available for this revision, no additional validated analytical data, full-series raw biological datasets, or independently traceable material suitable for the requested reanalysis were identified. Generating the missing evidence would require re-establishing the synthesis, analytical-characterization, and biological-testing workflows as a new prospective study. This practical boundary does not replace the missing evidence; it defines the narrow descriptive scope of the present retrospective analysis.

Taken together, the two computational layers answer different questions. The retrospective docking benchmark tests whether previously published target-level rankings translated into whole-cell phenotype, whereas the 2D descriptor analysis tests whether simple global molecular properties explain that phenotype. Neither layer alone accounts for the cellular ranking. This result supports the value of experimentally benchmarking molecular models rather than interpreting favorable in silico scores as direct surrogates for cellular potency. It also illustrates a practical role for retrospective model evaluation in drug discovery: computational prioritization can be assessed against subsequent experimental outcomes, the model’s limits can be made explicit, and subsequent modeling can be redirected toward properties not represented by a docking score alone. Future target prediction, exposure modeling, or pathway-network analyses may be useful for hypothesis generation. Still, they should be added only when complete input/output provenance can be retained and should remain clearly separated from direct mechanistic evidence.

## 5. Conclusions

This work provides a compound-level retrospective audit of previously published target-level molecular-modeling rankings against a subsequent MDA-MB-231/MCF-10A cellular dataset for the corresponding chemical series. Within the laboratory-reported data, compound **5e** had the lowest reported MDA-MB-231 IC_50_ and the largest descriptive SI, which is specific to this cell-line comparison. Prior docking rankings did not transfer directly to the cellular potency ranking, and no docking–phenotype association remained statistically significant after multiplicity correction. Because complete structural characterization, quantitative purity data, the complete full-series MTT raw dataset, orthogonal viability validation, sufficient biological replication, and an additional TNBC model are unavailable, the study does not establish identity-dependent SAR, generalized tumor selectivity, activity across TNBC as a disease class, apoptosis induction, a BCL-2 mechanism, or translational efficacy. The principal inference is methodological: prospective target-level molecular-model rankings and whole-cell phenotypic rankings are distinct endpoints that require direct experimental cross-validation. The secondary Annexin V/PI and RT-qPCR records are retained only in the Supplementary Information for transparency and do not contribute to this conclusion. Compound **5e** is therefore described only as the most active member of the available dataset and as a priority for future re-synthesis, complete analytical characterization, and independent prospective validation.

## Figures and Tables

**Table 1:** Experimentally evaluated compounds and recalculated theoretical molecular data.

Compound	R Substituent	Formula	MW (g/mol)	Exact Mass	[M + H]^+^ calc.
**5a**	phenyl	C_20_H_14_N_2_O_6_S	410.407	410.0573	411.0645
**5b**	4-Cl-phenyl	C_20_H_13_ClN_2_O_6_S	444.852	444.0183	445.0256
**5c**	4-Br-phenyl	C_20_H_13_BrN_2_O_6_S	489.303	487.9678	488.9750
**5d**	4-OH-phenyl	C_20_H_14_N_2_O_7_S	426.406	426.0522	427.0594
**5e**	4-F-phenyl	C_20_H_13_FN_2_O_6_S	428.397	428.0478	429.0551
**5f**	4-OCH_3_-phenyl	C_21_H_16_N_2_O_7_S	440.433	440.0678	441.0751
**5g**	4-CH_3_-phenyl	C_21_H_16_N_2_O_6_S	424.434	424.0729	425.0802
**5h**	4-NO_2_-phenyl	C_20_H_13_N_3_O_8_S	455.404	455.0423	456.0496
**5i**	2,4-Cl_2_-phenyl	C_20_H_12_Cl_2_N_2_O_6_S	479.297	477.9793	478.9866
**5j**	2,4-(OCH_3_)2-phenyl	C_22_H_18_N_2_O_8_S	470.459	470.0784	471.0857
**5k**	2-Cl-phenyl	C_20_H_13_ClN_2_O_6_S	444.852	444.0183	445.0256
**5l**	2-NO_2_-phenyl	C_20_H_13_N_3_O_8_S	455.404	455.0423	456.0496
**5m**	pyridin-4-yl	C_19_H_13_N_3_O_6_S	411.395	411.0525	412.0598
**5n**	indol-3-yl	C_22_H_15_N_3_O_6_S	449.444	449.0682	450.0754

Theoretical values were calculated from the structures assigned to the experimental sample labels. The structure-level cross-reference to the earlier computational library used for retrospective benchmarking is provided in Supplementary Information Table S1.

**Table 2:** Laboratory-reported IC_50_ values (mean ± SD; *n* = 2 independent experiments) from multi-concentration MTT measurements and descriptive selectivity indices.

Compound	MDA-MB-231 IC_50_ (µM)	MCF-10A IC_50_ (µM)	SI
**5a**	24.8 ± 0.5	94.2 ± 2.1	3.80
**5b**	25.0 ± 0.6	91.6 ± 1.8	3.66
**5c**	24.6 ± 0.4	89.8 ± 2.0	3.65
**5d**	22.1 ± 0.3	87.5 ± 1.7	3.96
**5e**	12.4 ± 0.2	96.1 ± 2.2	7.75
**5f**	20.8 ± 0.3	90.4 ± 1.9	4.35
**5g**	23.6 ± 0.4	86.9 ± 1.6	3.68
**5h**	14.8 ± 0.2	84.7 ± 1.5	5.72
**5i**	23.2 ± 0.4	88.6 ± 1.8	3.82
**5j**	23.8 ± 0.5	83.9 ± 1.4	3.53
**5k**	23.5 ± 0.3	85.8 ± 1.6	3.65
**5l**	24.9 ± 0.6	82.6 ± 1.3	3.32
**5m**	13.8 ± 0.3	93.4 ± 2.0	6.77
**5n**	24.2 ± 0.5	90.7 ± 1.7	3.75

SI = IC_50_(MCF-10A)/IC_50_(MDA-MB-231). The outsourced laboratory confirmed that each compound **5a**–**5n** underwent multi-concentration testing and that reported ± values represent SD between two independent measurements (*n* = 2). No compound-specific concentration-wise reports or complete full-series raw plate-reader export are available in the submitted records. Single-point viability values are not reported because the corresponding test concentration was not documented.

## Data Availability

The row-level numerical data used for the retrospective docking benchmark, rank-correlation summaries, leave-one-out sensitivity analysis, replicate-level RT-qPCR calculations, RNA quantification summary, primer documentation, Annexin V/PI source summary, and 2D descriptor analyses are provided in the Supplementary Information. No additional compound-specific concentration-wise MTT reports or complete full-series raw plate-reader files are available beyond the submitted numerical summaries. Complete original ^1^H/^13^C NMR records, validated HRMS metadata, quantitative purity measurements by HPLC/UPLC, qNMR, or equivalent methods, and additional biological replicates are not available for the full series. No missing raw values or analytical results were reconstructed or inferred.

## Supplementary Materials

The following supporting information can be downloaded at: https://www.mdpi.com/article/10.3390/biology15211999/s1, Table S1. Compound labels, assigned structures, theoretical molecular data, and prior-library cross-reference; Table S2. Canonical SMILES used as fixed molecular inputs; Table S3. Primary retrospective computational–experimental benchmark dataset; Table S4. Exploratory rank-correlation summary; Table S4A. Post hoc leave-one-out sensitivity analysis for the exploratory BCL-2 docking-versus-selectivity association; Table S4B. Annexin V/PI cell-population distribution in the available single independent experiment; Table S5. Facility Cq values and reproduced mean-Cq 2^−ΔΔCt calculations; Table S5A. Replicate-level RT-qPCR reanalysis based on two biological replicates identified by author clarification; Table S6. RNA concentration and spectrophotometric ratios; Table S7. Primer sequences used in the RT-qPCR assay; Table S8. Basic 2D physicochemical descriptor matrix for compounds **5a**–**5n**; Table S8A. Exploratory 2D descriptor–phenotype rank-correlation summary; Figure S1. Overlaid amplification profiles reproduced from the outsourced RT-qPCR facility report for CASP3, CASP8, CASP9, BCL2, BAX, and GAPDH across the analyzed samples; Figure S2. Gene-specific melt-curve outputs reproduced from the outsourced RT-qPCR facility report. Panels from top left to bottom right correspond to CASP3, CASP8, CASP9, BCL2, BAX, and GAPDH.

## Abbreviations

ADMET	absorption, distribution, metabolism, excretion, and toxicity
BAX	BCL2-associated X protein
BCL-2	B-cell lymphoma 2
EGFR	epidermal growth factor receptor
HER2	human epidermal growth factor receptor 2
IC_50_	half-maximal inhibitory concentration
MD	molecular dynamics
MTT	3-(4,5-dimethylthiazol-2-yl)-2,5-diphenyltetrazolium bromide
PARP-1	poly(ADP-ribose) polymerase-1
PI	propidium iodide
RT-qPCR	reverse-transcription quantitative polymerase chain reaction
SI	selectivity index
TNBC	triple-negative breast cancer
