# Supplementary material for: Retrospective Benchmarking of Previously Modeled Coumarin–Pyrimidine Structures Against MDA-MB-231/MCF-10A Cellular Activity
Source: Biology (Basel). 2026 Nov 4;15(21):1999. doi: 10.3390/biology15211999 (PMC13762295; doi:10.3390/biology15211999)
Supplement: Supplementary file 1 [file biology-15-01999-s001.zip › biology-4607171-supplementary.pdf]

Supplementary Information

Retrospective Benchmarking of Previously Modeled Coumarin–Pyrimidine Structures against MDA-MB-231/MCF-10A Cellular Activity

Analytical records and limitations. The available analytical material comprises positive-ion ESI mass-spectrum PDFs for intermediate 3 and compound labels 5a–5m, together with a partial ^{13}C NMR record for 5b. The complete mass-spectrometer/analyzer model, raw accurate-mass metadata, a complete original ^{1}H/^{13}C NMR series, and elemental analysis were not available for the full series. No quantitative purity measurements by HPLC/UPLC, qNMR, or an equivalent method were available in the study records. Structural characterization and quantitative purity are considered separately: spectroscopic and accurate-mass data address structural assignment, whereas HPLC/UPLC, qNMR, or another validated quantitative method can document sample purity. Because both areas are incompletely documented, identity-dependent and purity-qualified conclusions are limited accordingly. The author confirmed a file-label reversal between 5d and 5e and clarified that only page 1 (VB2, urea series) of the merged 5b ^{13}C PDF pertains to the present 5b compound; later VB18 pages belong to a guanidine-substituted series. The records are therefore provided as supporting analytical documentation rather than as complete validated HRMS/NMR characterization or quantitative purity evidence.

The supplementary tables and figures below accompany the revised Biology manuscript. Previously published docking values are reproduced only as prior predictions for retrospective cross-endpoint benchmarking; they are not presented as new docking results. The row-level data and summary statistics required to reconstruct the docking, descriptor, and RT-qPCR secondary analyses are consolidated in this file. The single-experiment Annexin V/PI record and the RT-qPCR records are included solely for transparency and are not used to support the primary conclusions. IC_{50} values are treated as laboratory-reported descriptive estimates, and structure-specific analyses use the compound assignments documented in the study records. The intended structures were previously disclosed in main-text Ref. [6]; the present work does not claim structural novelty or first synthesis on the basis of the available records.

Table S1. Compound labels, assigned structures, theoretical molecular data, and prior-library cross-reference

Compound	R substituent	Formula	MW (g/mol)	Exact mass	Calc. $[M+H]^+$	Prior-library label
5a	phenyl	$C_{20}H_{14}N_2O_6S$	410.407	410.0573	411.0645	5a
5b	4-chlorophenyl	$C_{20}H_{13}ClN_2O_6S$	444.852	444.0183	445.0256	5b
5c	4-bromophenyl	$C_{20}H_{13}BrN_2O_6S$	489.303	487.9678	488.9750	5c
5d	4-hydroxyphenyl	$C_{20}H_{14}N_2O_7S$	426.406	426.0522	427.0594	5d
5e	4-fluorophenyl	$C_{20}H_{13}FN_2O_6S$	428.397	428.0478	429.0551	5e
5f	4-methoxyphenyl	$C_{21}H_{16}N_2O_7S$	440.433	440.0678	441.0751	5j
5g	4-methylphenyl	$C_{21}H_{16}N_2O_6S$	424.434	424.0729	425.0802	5i
5h	4-nitrophenyl	$C_{20}H_{13}N_3O_8S$	455.404	455.0423	456.0496	5g
5i	2,4-dichlorophenyl	$C_{20}H_{12}Cl_2N_2O_6S$	479.297	477.9793	478.9866	5q
5j	2,4-dimethoxyphenyl	$C_{22}H_{18}N_2O_8S$	470.459	470.0784	471.0857	5o
5k	2-chlorophenyl	$C_{20}H_{13}ClN_2O_6S$	444.852	444.0183	445.0256	5k
5l	2-nitrophenyl	$C_{20}H_{13}N_3O_8S$	455.404	455.0423	456.0496	5l
5m	pyridin-4-yl	$C_{19}H_{13}N_3O_6S$	411.395	411.0525	412.0598	6e
5n	indol-3-yl	$C_{22}H_{15}N_3O_6S$	449.444	449.0682	450.0754	6f*

* Compound 5n is retained only as a sensitivity mapping to the intended indole-analogue entry in the prior library because the printed prior structure/SMILES is inconsistent with the structure assigned to 5n in the present study.

Table S2. Canonical SMILES used as fixed molecular inputs

Compound	Canonical SMILES
5a	Oc1nc(-c2ccccc2)cc(-c2c(OS(C)(=O)=O)c3ccccc3oc2=O)n1
5b	Oc1nc(-c2ccc(Cl)cc2)cc(-c2c(OS(C)(=O)=O)c3ccccc3oc2=O)n1
5c	Oc1nc(-c2ccc(Br)cc2)cc(-c2c(OS(C)(=O)=O)c3ccccc3oc2=O)n1
5d	Oc1nc(-c2ccc(O)cc2)cc(-c2c(OS(C)(=O)=O)c3ccccc3oc2=O)n1
5e	Oc1nc(-c2ccc(F)cc2)cc(-c2c(OS(C)(=O)=O)c3ccccc3oc2=O)n1
5f	COc1ccc(-c2cc(-c3c(OS(C)(=O)=O)c4ccccc4oc3=O)nc(O)n2)cc1
5g	Cc1ccc(-c2cc(-c3c(OS(C)(=O)=O)c4ccccc4oc3=O)nc(O)n2)cc1
5h	O=[N+]([O-])c1ccc(-c2cc(-c3c(OS(C)(=O)=O)c4ccccc4oc3=O)nc(O)n2)cc1
5i	Oc1nc(-c2ccc(Cl)cc2Cl)cc(-c2c(OS(C)(=O)=O)c3ccccc3oc2=O)n1
5j	COc1ccc(OC)c(-c2cc(-c3c(OS(C)(=O)=O)c4ccccc4oc3=O)nc(O)n2)c1
5k	Oc1nc(-c2ccccc2Cl)cc(-c2c(OS(C)(=O)=O)c3ccccc3oc2=O)n1
5l	O=[N+]([O-])c1ccccc1-c1cc(-c2c(OS(C)(=O)=O)c3ccccc3oc2=O)nc(O)n1
5m	Oc1nc(-c2ccncc2)cc(-c2c(OS(C)(=O)=O)c3ccccc3oc2=O)n1
5n	Oc1nc(-c2c[nH]c3ccccc23)cc(-c2c(OS(C)(=O)=O)c3ccccc3oc2=O)n1

Table S3. Primary retrospective computational–experimental benchmark dataset

The outsourced laboratory confirmed that all 5a–5n IC_{50} values derive from multi-concentration measurements and two independent experiments. No compound-specific concentration-wise reports or complete full-series raw plate-reader export are available in the submitted records. The IC_{50} values are therefore treated as laboratory-reported descriptive estimates and are not independently refitted from a complete raw dataset. Docking values are unchanged prior predictions from the Chemical Papers study.

Compound	Prior-library label	MDA-MB-231 IC_{50} (μM)	MCF-10A IC_{50} (μM)	SI	PARP-1 Vina score (kcal/mol)	EGFR Vina score (kcal/mol)	HER2 Vina score (kcal/mol)	BCL-2 Vina score (kcal/mol)	Mean docking rank	Potency rank
5a	5a	24.8	94.2	3.80	-7.6	-6.9	-9.1	-7.3	10.000	11
5b	5b	25.0	91.6	3.66	-7.6	-9.5	-9.6	-7.8	4.000	13
5c	5c	24.6	89.8	3.65	-7.6	-8.5	-9.3	-7.4	8.500	10
5d	5d	22.1	87.5	3.96	-7.5	-9.5	-9.3	-7.5	6.625	5
5e	5e	12.4	96.1	7.75	-7.7	-9.4	-9.5	-7.8	4.250	1
5f	5j	20.8	90.4	4.35	-7.3	-9.9	-8.4	-7.4	8.500	4
5g	5i	23.6	86.9	3.68	-7.7	-9.1	-9.5	-7.5	5.625	8
5h	5g	14.8	84.7	5.72	-7.3	-9.4	-9.3	-7.8	7.000	3
5i	5q	23.2	88.6	3.82	-7.5	-9.0	-8.8	-7.5	9.125	6
5j	5o	23.8	83.9	3.53	-7.1	-9.5	-7.8	-6.9	11.000	9
5k	5k	23.5	85.8	3.65	-7.7	-9.9	-8.6	-7.3	6.875	7
5l	5l	24.9	82.6	3.32	-7.7	-9.8	-9.1	-7.4	6.000	12
5m	6e	13.8	93.4	6.77	-8.6	-9.2	-9.5	-8.1	3.500	2

Table S4. Exploratory rank-correlation summary

Comparison	n	Spearman ρ	nominal p	BH q	Kendall τb	Kendall p
PARP-1 docking vs MDA-MB-231 IC_{50}	13	0.017	0.956	0.956	-0.041	0.851
EGFR docking vs MDA-MB-231 IC_{50}	13	0.042	0.893	0.956	0.066	0.758
HER2 docking vs MDA-MB-231 IC_{50}	13	0.081	0.793	0.956	0.094	0.664
BCL-2 docking vs MDA-MB-231 IC_{50}	13	0.492	0.087	0.393	0.384	0.081
PARP-1 docking vs SI	13	-0.039	0.898	0.956	0.069	0.754
EGFR docking vs SI	13	0.166	0.587	0.956	0.119	0.579
HER2 docking vs SI	13	-0.351	0.240	0.721	-0.255	0.239
BCL-2 docking vs SI	13	-0.680	0.011	0.095	-0.522	0.018
Mean docking rank vs potency rank	13	0.204	0.505	0.956	0.142	0.501

All nine comparisons are retrospective and hypothesis-generating. The BCL-2 docking-versus-SI association ($\rho = -0.680$; nominal $p = 0.011$) did not meet $q < 0.05$ after Benjamini–Hochberg adjustment ($q = 0.095$). Table S4A provides a post hoc leave-one-out robustness analysis for the BCL-2 docking-versus-SI signal.

Table S4A. Post hoc leave-one-out sensitivity analysis for the exploratory BCL-2 docking-versus-selectivity association

Compound omitted	Spearman ρ	Nominal p
5a	-0.7430	0.0056
5b	-0.7688	0.0035
5c	-0.6727	0.0165
5d	-0.6727	0.0165
5e	-0.6300	0.0281
5f	-0.7368	0.0063
5g	-0.7083	0.0099
5h	-0.6193	0.0318
5i	-0.6727	0.0165
5j	-0.6191	0.0318
5k	-0.6501	0.0221
5l	-0.6976	0.0117
5m	-0.6048	0.0372

The inverse BCL-2 docking-versus-SI association retained its direction after omission of every single compound (Spearman ρ range, −0.769 to −0.605). This post hoc robustness analysis does not alter the primary Benjamini–Hochberg-adjusted result ($q = 0.095$) and is not interpreted as confirmatory evidence.

Exploratory secondary biological records

Annexin V-FITC/PI source record

MDA-MB-231 cells were seeded at 0.5×10^6 cells/well in 6-well plates and incubated overnight. Cells were exposed for 24 h to compound 5e at a nominal concentration of 12.4 µM, with the DMSO vehicle as the negative control and doxorubicin (1 µM) as the positive control. The exact final DMSO concentration was not independently documented. After treatment, cells were harvested, washed, resuspended in Annexin V binding buffer, stained with 5 µL FITC-conjugated Annexin V for 15 min at room temperature in the dark, followed by 5 µL propidium iodide and 400 µL of 1× binding buffer, and analyzed on a BD Accuri C6 Plus flow cytometer with at least 15,000 events acquired per condition. The available record represents one independent experiment. It is provided solely as a descriptive source record and is not treated as evidence that 5e induces apoptosis (see main-text Ref. [13] for methodological context).

Table S4B. Annexin V/PI cell-population distribution in the available single independent experiment.

**Condition**	**Necrotic (%)**	**Late apoptotic (%)**	**Viable (%)**	**Early apoptotic (%)**	**Total apoptotic (%)**
DMSO vehicle control*	0.83	15.41	71.97	11.79	27.20
Doxorubicin control	0.04	89.99	7.84	2.13	92.12
5e	4.89	26.29	55.14	13.69	39.98

Total apoptotic = early + late apoptotic. *The source output labeled this condition “Untreated”; subsequent author clarification identified it as the DMSO vehicle control. Combined apoptosis was 39.98% after 5e and 27.20% in the vehicle control, an absolute difference of 12.78 percentage points. Because the control background was substantial and only one independent experiment is available, no inferential statistics are applied and no apoptosis-effect claim is made.

RT-qPCR methods, source data, and secondary calculations

MDA-MB-231 cells were treated for 24 h with 5e at a nominal concentration of 12.4 µM or with doxorubicin (1 µM); untreated cells served as the calibrator. Total RNA was isolated using RNA-ISO Plus reagent according to the laboratory protocol. The RNA concentrations and spectrophotometric ratios are reported in Table S6. The suboptimal A_{260}/A_{280} and A_{260}/A_{230} values are retained transparently. No archived record documents RNA re-purification or a formal inhibition assessment.

The outsourced facility report records iScript cDNA synthesis with an RNA input of 800 ng. A separate manuscript record reports a reverse-transcription program of 25 °C for 5 min, 46 °C for 20 min, and 95 °C for 1 min. Gene-specific primers targeted CASP3, CASP8, CASP9, BCL2, and BAX, with GAPDH as the reference gene; sequences are provided in Table S7 (see main-text Refs. [14–16] for methodological context).

Relative quantification was performed using SYBR Green chemistry on a QuantStudio 3 Real-Time PCR System and the 2^−ΔΔCt method, with GAPDH as the reference transcript and untreated cells as the calibrator. The two supplied Cq values per condition were identified by author clarification as two biological replicates; no separate technical-replicate layer is encoded. The final DMSO concentration and vehicle matching of the calibrator cannot be independently verified. A complete master-mix identity, thermal-cycling record, amplification-efficiency dataset, and formal inhibition assessment were unavailable and are not reconstructed. These data are descriptive and are not used to establish apoptosis, pathway activation, or BCL-2 engagement.

The supplied facility workbook contains two Cq values for each condition–gene combination. Table S5 reproduces the facility calculations, and Table S5A provides a replicate-level ΔCt/2^−ΔΔCt reanalysis using the Cq1/Cq2 pairing as organized in the workbook. Means ± SD are descriptive summaries of n = 2 biological replicates and are not used for inferential statistics. Doxorubicin (1 µM, 24 h) was the standard-control condition. The facility workbook identifies untreated cells as the calibrator; because the final DMSO concentration of the 5e treatment was not documented, vehicle matching cannot be independently verified. A complete thermal-cycling record, amplification-efficiency dataset, and formal inhibition assessment were not available and are not reconstructed.

Table S5. Facility Cq values and reproduced mean-Cq 2^−ΔΔCt calculations

Gene	Condition	Cq 1	Cq 2	Mean Cq	Mean GAPDH Cq	ΔCt	ΔΔCt	Fold change
CASP3	Untreated	25.725	25.781	25.7530	16.8445	8.9085	0.0000	1.0000
CASP3	Doxorubicin control	22.173	22.089	22.1310	16.8640	5.2670	-3.6415	12.4800
CASP3	5e	22.819	23.087	22.9530	16.8705	6.0825	-2.8260	7.0910
CASP8	Untreated	25.127	25.084	25.1055	16.8445	8.2610	0.0000	1.0000
CASP8	Doxorubicin control	21.713	21.751	21.7320	16.8640	4.8680	-3.3930	10.5050
CASP8	5e	23.052	23.118	23.0850	16.8705	6.2145	-2.0465	4.1310
CASP9	Untreated	23.078	23.256	23.1670	16.8445	6.3225	0.0000	1.0000
CASP9	Doxorubicin control	21.082	21.174	21.1280	16.8640	4.2640	-2.0585	4.1660
CASP9	5e	22.137	22.168	22.1525	16.8705	5.2820	-1.0405	2.0570
BCL2	Untreated	21.715	21.093	21.4040	16.8445	4.5595	0.0000	1.0000
BCL2	Doxorubicin control	25.472	25.517	25.4945	16.8640	8.6305	4.0710	0.0595
BCL2	5e	25.718	25.725	25.7215	16.8705	8.8510	4.2915	0.0511
BAX	Untreated	21.419	21.357	21.3880	16.8445	4.5435	0.0000	1.0000
BAX	Doxorubicin control	18.824	18.913	18.8685	16.8640	2.0045	-2.5390	5.8120
BAX	5e	19.453	19.409	19.4310	16.8705	2.5605	-1.9830	3.9530

Table S5A. Replicate-level RT-qPCR reanalysis based on two biological replicates identified by author clarification

Gene	Condition	Fold Rep 1	Fold Rep 2	Mean fold	SD fold
CASP3	Untreated	0.978	1.023	1.000	0.032
CASP3	Doxorubicin control	12.223	12.742	12.482	0.367
CASP3	5e	7.555	6.655	7.105	0.636
CASP8	Untreated	0.945	1.058	1.002	0.080
CASP8	Doxorubicin control	10.733	10.282	10.507	0.319
CASP8	5e	4.104	4.158	4.131	0.038
CASP9	Untreated	1.020	0.980	1.000	0.028
CASP9	Doxorubicin control	4.336	4.001	4.169	0.237
CASP9	5e	2.019	2.096	2.057	0.054
BCL2	Untreated	0.773	1.294	1.033	0.368
BCL2	Doxorubicin control	0.061	0.058	0.060	0.002
BCL2	5e	0.050	0.052	0.051	0.002
BAX	Untreated	0.939	1.065	1.002	0.090
BAX	Doxorubicin control	6.044	5.589	5.816	0.322
BAX	5e	3.780	4.134	3.957	0.250

Replicate-level fold values were calculated as $2^{-(\Delta Ct\ replicate\ -\ mean\ untreated\ \Delta Ct\ for\ the\ corresponding\ gene)}$, using Cq1/Cq2 pairing as organized in the facility workbook. Mean and SD are descriptive summaries of $n = 2$ biological replicates and are not used for inferential statistics. The facility point estimates, obtained by averaging Cq before ΔCt/ΔΔCt calculation, are retained for comparison and are closely concordant with the replicate-level central estimates.

Table S6. RNA concentration and spectrophotometric ratios

Condition	RNA (ng/µL)	A_{260}/A_{280}	A_{260}/A_{230}
Untreated	1825.71	1.73	1.15
Doxorubicin control	1757.09	1.68	1.26
5e	1803.58	1.71	1.09

These measurements were taken from the RNA quantification record dated 27 April 2026. The spectrophotometric purity ratios are suboptimal and are therefore disclosed as a limitation in the main manuscript.

Table S7. Primer sequences used in the RT-qPCR assay

Gene	Accession	Forward primer (5′–3′)	Reverse primer (5′–3′)	Tm F (°C)	Tm R (°C)
CASP3	NM_001354781.2	AGGACTCTAGACGGCATCCA	CCAGTGAGACTTGGTGCAGT	59.74	59.89
BCL2	NM_000633.3	GGGGTCATGTGTGTGGAGAG	CATCCCAGCCTCCGTTATCC	60.04	59.96
CASP9	NM_001229	GCACTGGCTCCAACATCGA	CATGAAATGCAGCGAGGAGAA	59.50	59.40
BAX	NM_004324	TCAGGATGCGTCCACCAAGAAG	TGTGTCCACGGCGGCAATCATC	60.35	59.99
CASP8	NM_001080125	AGAAGAGGGTCATCCTGGGAGA	TCAGGACTTCCTTCAAGGCTGC	60.08	60.00
GAPDH	NM_002046.7	GAAGGTGAAGGTCGGAGT	GAAGATGGTGATGGGATTTC	58.13	59.93

Figure S1. RT-qPCR amplification profiles

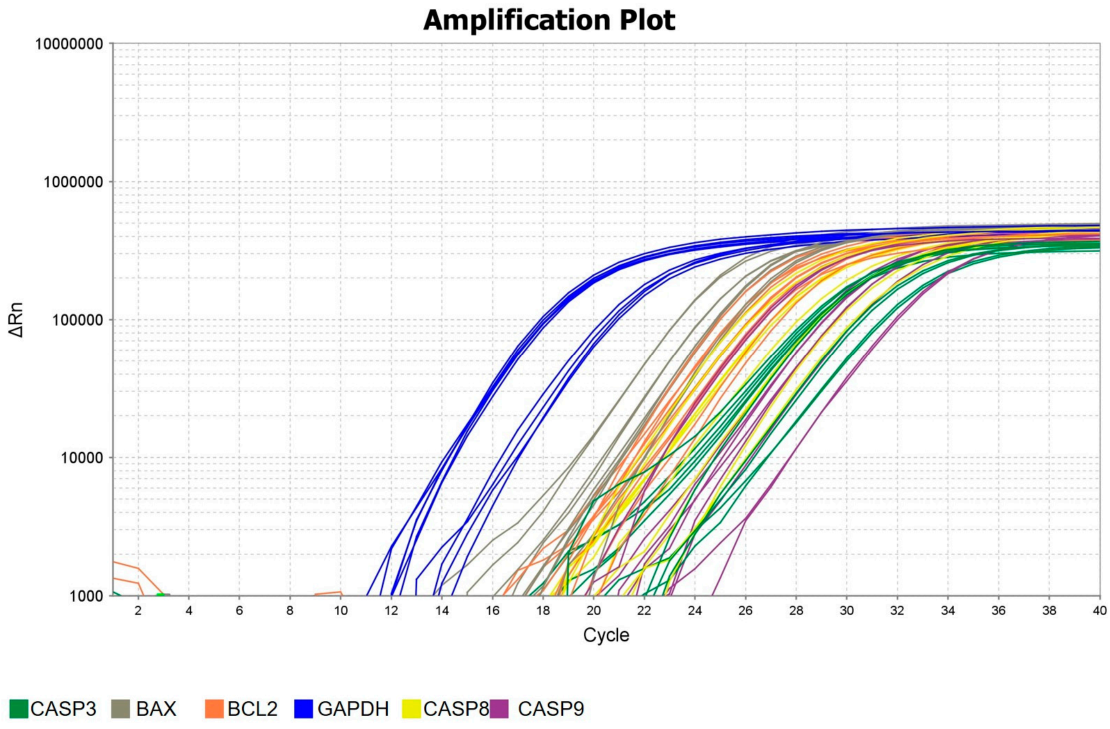

Figure S1. Overlaid amplification profiles reproduced from the outsourced RT-qPCR facility report for CASP3, CASP8, CASP9, BCL2, BAX, and GAPDH across the analyzed samples. The exported image identifies assays by gene but does not encode condition-level curve identities.

Figure S2. Gene-specific melt-curve profiles

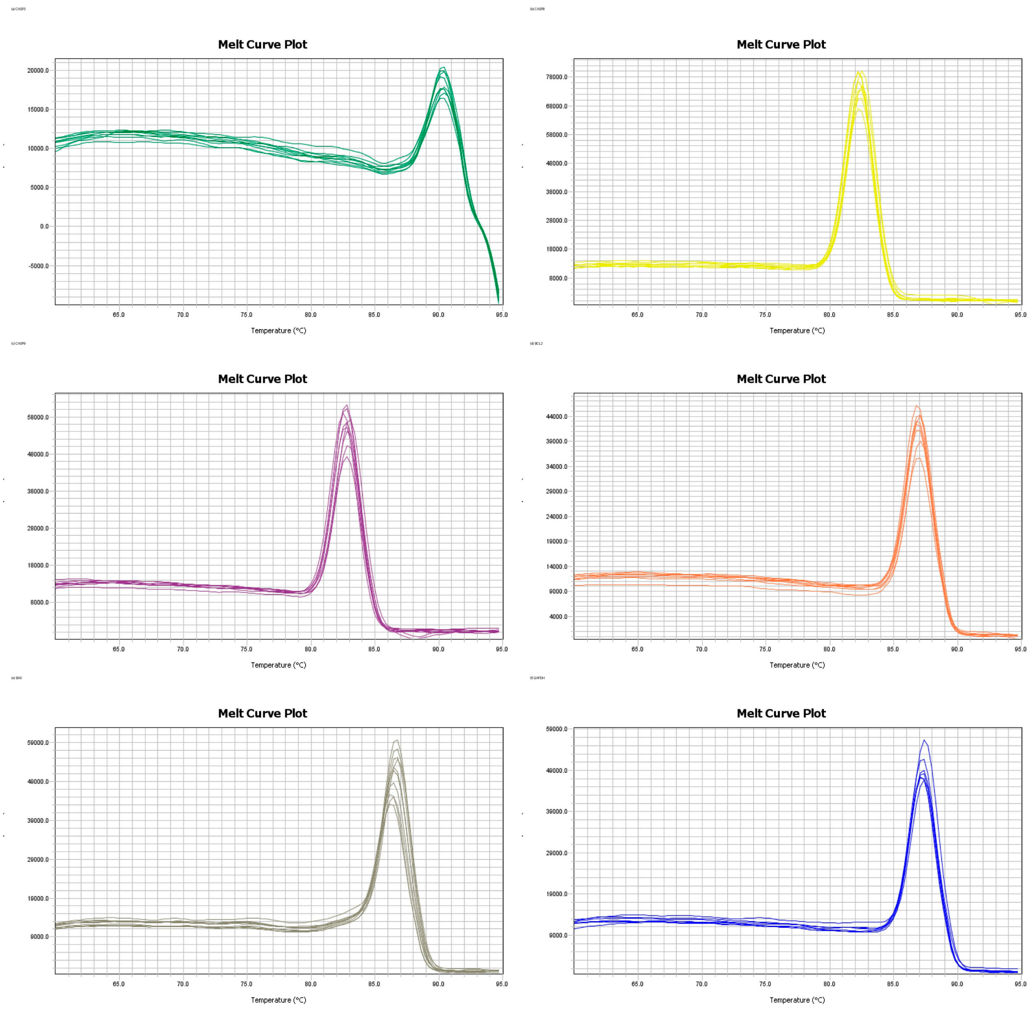

Figure S2. Gene-specific melt-curve outputs reproduced from the outsourced RT-qPCR facility report. Panels from top left to bottom right correspond to CASP3, CASP8, CASP9, BCL2, BAX, and GAPDH. Each assay displays one predominant melting transition in the provided output; these plots support a predominant amplification product but do not substitute for sequence-level amplicon validation or formal amplification-efficiency assessment.

Two-dimensional physicochemical descriptor analysis

Basic RDKit descriptors (MW, cLogP, TPSA, HBA, HBD, rotatable-bond count, fractionCSP3, and aromatic-ring count) were calculated for all 14 compounds using RDKit 2025.09.4. Table S8 provides the descriptor matrix and Table S8A summarizes the 16 exploratory descriptor–phenotype rank correlations. No descriptor showed a compelling monotonic association with MDA-MB-231 IC_{50} or SI after Benjamini–Hochberg adjustment; the largest nominal trend was MW versus SI (Spearman $\rho = -0.506$, $p = 0.065$).

Reproducibility of secondary analyses

The retrospective benchmark can be reconstructed from Tables S1, S3, S4, and S4A using the rank-based procedures specified in the main Methods. The Annexin V/PI source summary is provided in Table S4B. The descriptor analysis can be reconstructed from Tables S8 and S8A, and the RT-qPCR secondary analysis from Tables S5 and S5A. Software versions used for the reported secondary analyses are stated in the main manuscript.

Table S8. Basic 2D physicochemical descriptor matrix for compounds 5a–5n

Compound	MW	cLogP	TPSA	HBA	HBD	RotB	FractionCSP3	Aromatic rings
5a	410.407	2.961	119.59	8	1	4	0.050	4
5b	444.852	3.614	119.59	8	1	4	0.050	4
5c	489.303	3.724	119.59	8	1	4	0.050	4
5d	426.406	2.667	139.82	9	2	4	0.050	4
5e	428.397	3.100	119.59	8	1	4	0.050	4
5f	440.433	2.970	128.82	9	1	5	0.095	4
5g	424.434	3.269	119.59	8	1	4	0.095	4
5h	455.404	2.869	162.73	10	1	5	0.050	4
5i	479.297	4.268	119.59	8	1	4	0.050	4
5j	470.459	2.978	138.05	10	1	6	0.136	4
5k	444.852	3.614	119.59	8	1	4	0.050	4
5l	455.404	2.869	162.73	10	1	5	0.050	4
5m	411.395	2.356	132.48	9	1	4	0.053	4
5n	449.444	3.442	135.38	8	2	4	0.045	5

Table S8A. Exploratory 2D descriptor–phenotype rank-correlation summary

Outcome	Descriptor	n	Spearman ρ	Nominal p	BH q	Kendall τb	Kendall p
MDA-MB-231 IC_{50}	MW	14	0.273	0.345	1.000	0.177	0.380
MDA-MB-231 IC_{50}	cLogP	14	0.350	0.220	1.000	0.221	0.273
MDA-MB-231 IC_{50}	TPSA	14	-0.125	0.671	1.000	-0.114	0.596
MDA-MB-231 IC_{50}	HBA	14	-0.207	0.478	1.000	-0.181	0.421
MDA-MB-231 IC_{50}	HBD	14	0.000	1.000	1.000	0.000	1.000
MDA-MB-231 IC_{50}	RotB	14	-0.019	0.948	1.000	-0.016	0.944
MDA-MB-231 IC_{50}	FractionCSP3	14	-0.244	0.401	1.000	-0.143	0.519
MDA-MB-231 IC_{50}	AromaticRings	14	0.172	0.557	1.000	0.145	0.535
Selectivity index	MW	14	-0.506	0.065	1.000	-0.331	0.100
Selectivity index	cLogP	14	-0.370	0.193	1.000	-0.265	0.188
Selectivity index	TPSA	14	0.005	0.987	1.000	0.013	0.953
Selectivity index	HBA	14	-0.002	0.993	1.000	0.042	0.853
Selectivity index	HBD	14	0.101	0.730	1.000	0.086	0.715
Selectivity index	RotB	14	-0.205	0.482	1.000	-0.144	0.530
Selectivity index	FractionCSP3	14	-0.005	0.986	1.000	0.000	1.000
Selectivity index	AromaticRings	14	-0.034	0.907	1.000	-0.029	0.901

BH q values are Benjamini–Hochberg adjusted across the 16 descriptor–phenotype comparisons. Values are exploratory and are not interpreted as a predictive QSAR model.

Available analytical records

Positive-ion ESI mass-spectrum PDFs were supplied for intermediate 3 and sample labels 5a–5m, together with one merged ^{13}C NMR record labelled 5b. The facility was reported to use a Waters ACQUITY platform with MassLynx software in positive-ion ESI mode, but the complete analyzer/model, calibration/error information, and raw accurate-mass metadata were unavailable. These files are therefore not described as HRMS. No complete original ^{1}H/^{13}C NMR series, elemental analysis, HPLC/UPLC, qNMR, or other quantitative purity records are available. The analytical records are summarized here to define the evidentiary limits of the chemical dataset and are not used as complete structure- or purity-confirmation evidence.

The author confirmed that the files supplied as 5d and 5e had been exchanged; the supplied 5e..pdf/VB5 record (m/z 427.09) therefore corresponds to the sample labelled 5d, and the supplied 5d..pdf/VB4 record (m/z 429.05) to the sample labelled 5e. Other supplied ESI-MS records include inconsistent, non-discriminating, or duplicate-appearing traces and are not used as independent structure-confirmation evidence. No independently labelled 5n spectrum or additional VB-number-to-compound cross-reference was available.

The merged 5b ^{13}C NMR PDF was clarified by the author: page 1 identifies VB2_13C and belongs to the urea series used in the present study, whereas later pages marked VB18_13C belong to a guanidine-substituted series. Only page 1 is therefore relevant to the present 5b sample. No original raw/FID record, additional 5b spectrum, or original 5k ^{13}C spectrum was available. The complete ^{13}C assignments from the original draft are not reproduced as validated structure-confirmation data.
